# Regulation by Light of Chemotaxis to Nitrite during the Sexual Life Cycle in *Chlamydomonas reinhardtii*

**DOI:** 10.3390/plants3010113

**Published:** 2014-02-26

**Authors:** Elena Ermilova, Zhanneta Zalutskaya

**Affiliations:** Laboratory of Adaptation in Microorganisms, Saint-Petersburg State University, Oranienbaumskoe Schosse 2, Stary Peterhof, Saint-Petersburg 198504, Russia; E-Mail: z.zalutskaya@bio.spbu.ru

**Keywords:** chemotaxis, *Chlamydomonas*, phototropin, protein kinases

## Abstract

Nitrite plays an important role in the nitrogen metabolism of most cells, including *Chlamydomonas reinhardtii.* We have shown that vegetative cells of *C. reinhardtii* are attracted by nitrite. The *Nia1nit2* mutant with defects in genes encoding the nitrate reductase and regulatory protein NIT2 respectively was found to exhibit normal chemotaxis to nitrite. The data suggest that chemotaxis events appear to be specific and independent of those involved in nitrate assimilation. Unlike vegetative cells and noncompetent pregametes, mature gametes did not show chemotaxis to nitrite. Just like gamete formation, the change in chemotaxis mode is controlled by the sequential action of two environmental cues, removal of nitrogen from the medium and light. Comparative analysis of wild-type and RNAi strains with reduced level of phototropin has indicated that switch-off of chemotaxis towards nitrite is dependent on phototropin. The studies revealed individual elements of the phototropin-dependent signal transduction pathway involved in the blue-light-controlled change in chemotaxis mode of *C. reinhardtii* during gamete formation: three protein kinases, one operating against signal flux and two that promote signal transduction. We have proposed a working model for the signaling pathway by which blue light controls chemotaxis towards attractants, which are nitrogen sources, during pregamete-to-gamete conversion of *C. reinhardtii*.

## 1. Introduction

Algae use light not only as a source of energy for photosynthesis but also as a source of environmental information to regulate a variety of processes, including pigment biosynthesis, chloroplast movement, circadian rhythms, transport activity, cell cycle, motile behaviour and development [1,2,3,4,5,6,7,8,9]. One of these phenomena, the differentiation of vegetative cells into sexually mature cells has been widely studied in *Chlamydomonas* and may proceed in two steps [10]. The depletion of a usable nitrogen source induces sexually incompetent gametes, named pregametes in the dark [11]. In a second step, these pregametes may be converted into mating-competent gametes by light irradiation [12]. The flavin-based blue light receptor phototropin (Phot) in *C. reinhardtii* controls the progression of the sexual life cycle of this green alga [6]. Phot in *C*. *reinhardtii* also functions in transcriptional regulation [13] and in the control of phototaxis that desensitizes the eyespot when blue light intensities increase [14]. The interacting proteins of Phot involved in downstream signaling are not known in this alga. Analysis of the signaling pathway by which light controls the conversion of pregametes to gametes using pharmacological compounds suggests the participation of protein kinases and cAMP in this signaling cascade [15]. Thus, a protein kinase C (PKC)-like activity operates against signal flux while a protein tyrosine kinase (PTK) stimulates signal flux. The intracellular accumulation of cAMP, either by feeding or by the use of phosphodiesterase inhibitors, inhibited the light-dependent conversion of pregametes to gametes suggesting a role of this cyclic nucleotide as a negative regulator.

In *C. reinhardtii,* vegetative cells, pregametes and mature gametes are motile. Vegetative cells and mating incompetent pregametes exhibit chemotaxis towards ammonium and nitrate [2,16,17]. Chemotaxis to these ions enables vegetative cells to orient themselves towards the nitrogen sources. Gametic differentiation has been shown to be associated with changes not only in the cells’ biochemistry and subcellular morphology [10] but also in chemotactic behavior [2]. Mature gametes are not attracted by ammonium and nitrate [2,17]. Like the development of mating competence, the loss of chemotaxis requires the action of two environmental signals: lack of a nitrogen source and blue light. We have shown that the photoreceptor, which mediates the loss of chemotaxis to these attractants during gametogenesis, is Phot [17,18]. Ammonium, nitrate and another nitrogen source, nitrite, also play an important role in sexual differentiation of *C. reinhardtii* [19]. Vegetative cells only in the absence of these nitrogen sources initiate the program of sexual differentiation [12,20]. We suggest that loss of chemotaxis towards nitrogen sources by gametes may be one of prerequisites to their successful mating at locations accessible to light. However, chemotactic behaviour to nitrite has not been characterized. Therefore, the goals of this study were to (1) test chemotaxis to nitrite during sexual life cycle; (2) analyze the involvement of Phot in control of chemotaxis to nitrite; and (3) reveal individual elements of the signal transduction pathway involved in the blue-light-controlled change in chemotaxis to nitrite and nitrate. For the analyses, we used RNAi strains with reduced levels of phototropin, chemotaxis assay and pharmacological compounds known to target defined signaling steps. Our results demonstrate that vegetative cells and pregametes of *C. reinhardtii* are attracted by nitrite and signals generated by activation of phototropin feeds into a linear signal chain that switches off chemotaxis towards ammonium, nitrate and nitrite.

## 2. Results

### 2.1. Nitrite as Chemoattractant

**Figure 1:**
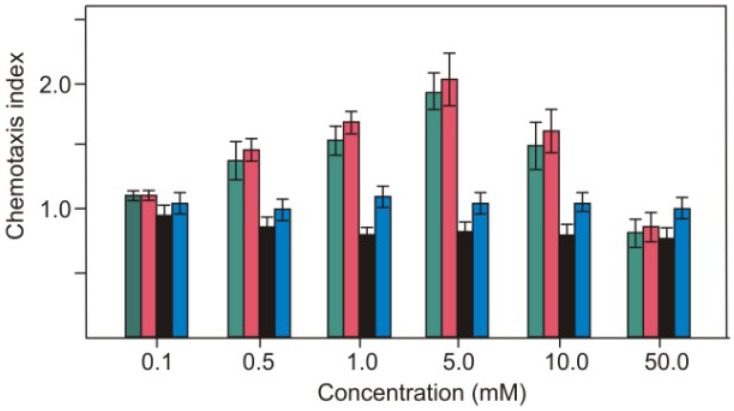
Reaction of vegetative cells of strain CC-124 to nitrite (KNO_2_) (■), nitrate (KNO_3_) (■), KCl (■) and KH_2_PO_4_ (■). Data are means ± SD of 5 independent experiments.

Earlier experiments showed that *C. reinhardtii* vegetative cells were attracted to nitrate [17]. We wondered whether *C. reinhardtii* exhibits chemotaxis to nitrite. Strain CC-124 (*nia1, nit2*) accumulated in capillaries containing KNO_2_ (Figure 1). The peak accumulation of the cells was observed when the capillary contained 5 mM of KNO_2_. The threshold concentration (the lowest concentration needed to elicit an observable response) was approximately 0.5 mM. At the same time *C. reinhardtii* did not react chemotactically to KCl and KH_2_PO_4_. It means that nitrite but not potassium acts as an attractant for chemotactic response. 

**Figure 2:**
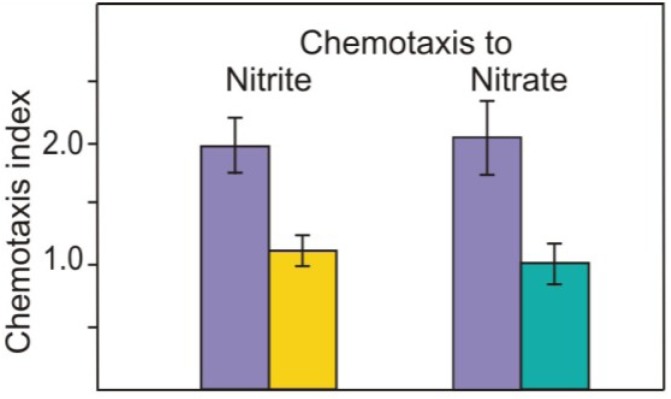
Effect of nitrate and nitrite to the chemotactic responses of CC-124 vegetative cells in different media. Vegetative cells in TAP-N (■); TAP-N supplied with nitrate (5 mM) (■), and TAP-N supplemented with nitrite (5 mM) (■) were assayed for chemotaxis to 5 mM nitrite or 5 mM nitrate as indicated. Data are means ± SD of 3 independent experiments.

We were surprised that the CC-124 strain, which does not express many of the genes required for nitrate/nitrite assimilation (*Nia1, Nrt2.1, Nrt2.2, Nii1, Nar2* and *Nar1.1*) [21], showed normal chemotactic responses both to nitrite and nitrate. These data suggest that nitrate assimilation is unlikely to play a role in mediating the chemotaxis to nitrate/nitrite. These results were supported by the competition tests when both media, in the bath and in the capillary, contained the second attractant of the same concentration (5 mM KNO_2_ or 5 mM KNO_3_) (Figure 2). Nitrate completely prevented chemotaxis to nitrite. Additionally chemotaxis to nitrate was also affected by nitrite. These data indicate that the same components may be shared in the control of chemotaxis to both anions nitrate and nitrite.

### 2.2. Role of Light in Change of Chemotaxis to Nitrite during Gametogenesis

The ability of unicellular organisms to differentiate in response to nutrient availability is essential to their survival in a changing environment [22]. The initial step in the sexual life cycle of *C. reinhardtii* is gametogenesis. During gametogenesis, vegetative cells are transformed into mating-competent gametes. Effective formation of mating ability depends on removal of nitrogen from the medium and on the presence of light [10].

**Figure 3:**
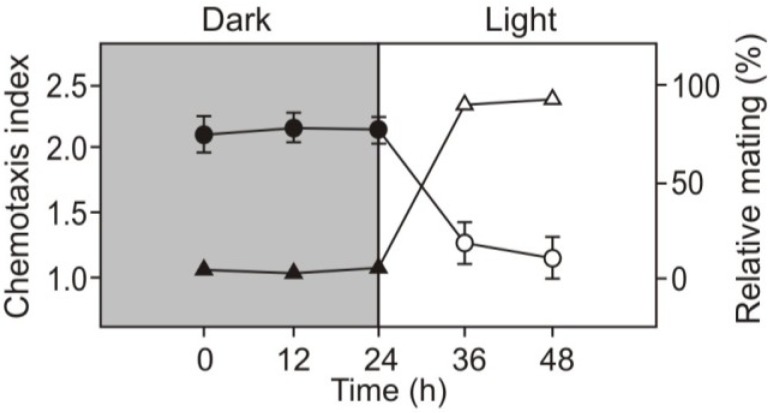
Chemotactic responses of pregametes (●) and gametes obtained from pregametes (○) towards 5 mM nitrite. Vegetative cells of *mt^−^* were resuspended in TAP-N at time 0. The percentage of mating (Δ) is given for a comparison. At the times indicated, cells were mixed with an excess of sexually competent mating partner, and incubated in the dark. After 1h, the cells were fixed by the addition of glutaraldehyde and relative mating was determined as described in the Experimental Section. Data are means ± SD of triplicate determination from a representative experiment.

We wondered next if pregametes and gametes react chemotactically to nitrite. Figure 3 shows that pregametes are attracted to nitrite. Even though acetate as source of carbon and energy was present in the nitrogen-free medium, cells were not capable to complete the program of differentiation and were not competent for mating. The results demonstrate that nitrogen deprivation is not enough to change the mode of chemotactic behavior to nitrite. Additionally the data indicate that light is not essential for chemotaxis towards nitrite.

Upon incubation in the light, pregametes are converted into mating-competent gametes [23]. We next tested whether these two environmental signals might also act on change of chemotaxis to nitrite sequentially. For these tests, pregametes, generated from vegetative cells by incubation in the dark without a utilizable nitrogen source, were exposed to light. As shown in Figure 3, light induced the switch-off of to chemotaxis in cells. Therefore, chemotactic behavior to nitrite may be differentiated according to the development of competence ability. We assume that chemotaxis towards nitrite, nitrate and ammonium in gametes can be blocked by using identical light-dependent signaling pathways. 

### 2.3. Effect of Reduced Phototropin Levels on the Kinetics of Changes in Chemotaxis to Nitrite

**Figure 4:**
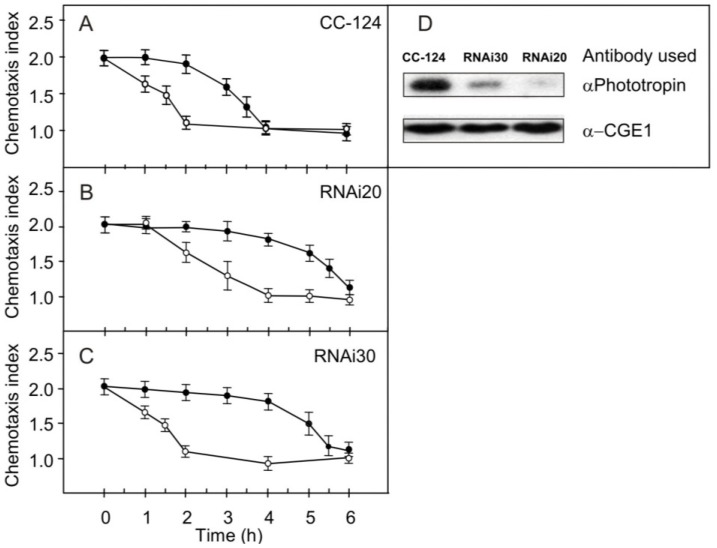
Chemotaxis of strains CC-124 (**A**); RNAi20 (**B**) and RNAi30 (**C**) to nitrite. Pregametes, generated from synchronously growing cells by incubation in TAP-N medium in the dark for 18 h, at time 0 were shifted into white light at the fluence rates of 60 µmol m^−2^ s^−1^ (○) and of 30 µmol m^−2^ s^−1^ (●). Relative amounts of phototropin in different transformants harbouring RNAi constructs as compared to the strain CC-124 (**D**).

Absorption of blue light by phototropin results in the activation of signaling pathways that control the switch-off of chemotaxis to ammonium and nitrate during gametogenesis [17,18]. RNA-interference strains with reduced levels of the blue-light receptor phototropin showed an attenuated inactivation of chemotaxis to these attractants. To determine if phototropin was involved in changes in chemotactic activity to nitrite, we tested behavior of two strains that exhibited reduced phototropin levels. The RNAi20 strain, having severely reduced phototropin levels [18], exhibited a switching-off of chemotaxis at a fluence rate of 60 μmol m^−2^ s^−1^, however, with a delay of about 2 h when compared to the parental wild-type strain CC-124 (Figure 4). 

At a fluence rate of 30 µmol m^−2^ s^−1^, the wild-type strain showed a distinct decrease in the chemotaxis index after 3 h in nitrogen-free medium; with the RNAi20 strain this was observed only after 5 h. At a fluence rate of 60 µmol m^−2^ s^−1^, the strain RNAi30 exhibited kinetics of loss of chemotaxis to nitrite that was essentially the same as those observed for the wild-type strain (Figure 4C). At a fluence rate of 30 µmol m^−2^ s^−1^, however, this strain showed a reduced loss of chemotaxis when compared to parental strain CC-124. As in the case of chemotactic behavior to ammonium, the kinetics observed for strain RNAi30 were faster than those for strain RNAi20 and thus were intermediate between those of the wild-type strain and RNAi20. As shown earlier [18], both strains exhibited reduced levels of phototropin, although the Phot levels of the RNAi20 strain were distinctly lower than those of RNAi30. Thus, the kinetics of the loss of chemotaxis to nitrite approximately correlates with the level of Phot protein detected by specific antibodies. We conclude that phototropin is the photoreceptor used by blue light to control the switch-off of chemotaxis in response to nitrite during gametogenesis. 

### 2.4. Effects of Pharmacological Compounds on the Kinetics of Changes in Chemotaxis to Nitrite in the Strain RNAi20

Analysis of the signaling pathway by which light controls the switch-off of chemotaxis to ammonium suggests the participation of protein kinases and cAMP in this signaling cascade [24]. To determine whether the switch-off of chemotaxis to ammonium and nitrite is controlled by identical key phototropin-controlled signaling components, we analyzed chelerythrine, staurosporine, papaverine, IBMX (3-isobutyl-1-methylxanthine) and db-cAMP, compounds shown to activate loss of chemotaxis to ammonium [24], for their ability to compensate for the slowed-down loss of chemotaxis seen in strain RNAi20.

**Figure 5:**
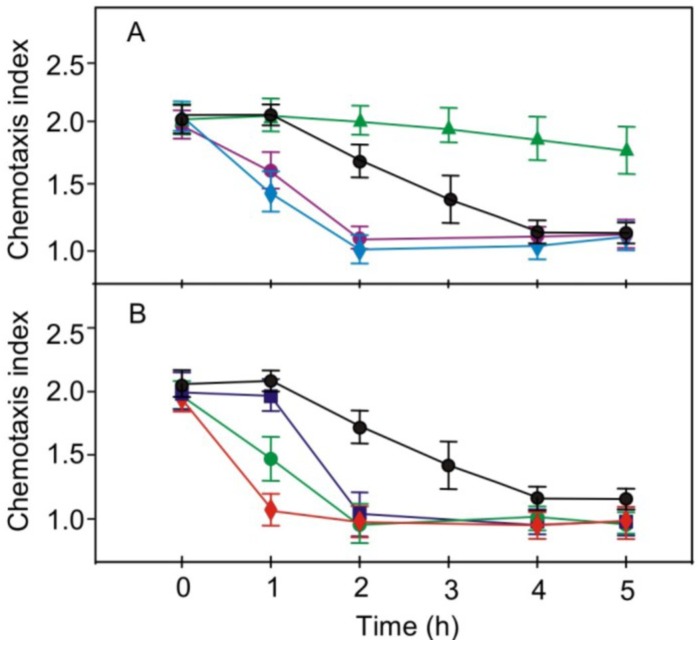
Effects of various pharmacological compounds on the light-induced loss of chemotaxis towards nitrite in pregametes of phototropin-reduced strain RNAi20. (**A**) Effect of staurosporine (♦), chelerythrine (●) or SC-9 (▲) on the loss of chemotaxis in pregametes of RNAi20 that were shifted into white light at time 0; (**B**) Treatment with papaverine (●), IBMX (♦) or db-cAMP (■) on the loss of chemotaxis in pregametes of RNAi20 that were shifted into white light at time 0. Compounds were added to pregametes 10 min prior to start of illumination. The concentrations used were 1 μM for chelerythrine, 50 μM for papaverine, 50 μM for IBMX, 20 nM for staurosporine and and 200 μM for SC-9. Non-treated cells (●) were used as control. The fluence rate was 60 µmol m^−2^ s^−1^.

Staurosporine at low concentrations is a specific inhibitor of PKC, as is chelerythrine. After addition of staurosporine or chelerythrine, the kinetics of loss of chemotaxis to nitrite in the light observed for strain RNAi20 were faster than those seen without treatment (Figure 5A). 

Both inhibitors at the concentrations used did not influence cellular motility (data not shown). Treatment of pregametes with the naphthalene sulfonamide SC-9, a PKC activator, specifically inhibited loss of chemotaxis to nitrite in the light (Figure 5A). The combined data suggest that a PKC-like component may be involved in signaling that results in the loss of chemotaxis to nitrite.

**Figure 6:**
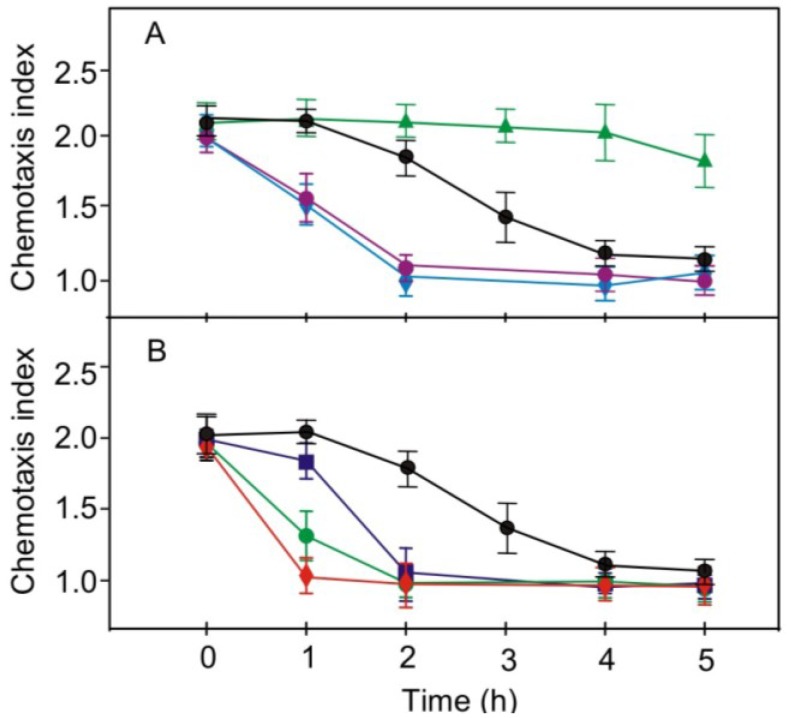
Effects of various pharmacological compounds on the light-induced loss of chemotaxis towards nitrate in pregametes of phototropin-reduced strain RNAi20. (**A**) Effect of staurosporine (♦), chelerythrine (●) or SC-9 (▲) on the loss of chemotaxis in pregametes of RNAi20 that were shifted into white light at time 0; (**B**) Treatment with papaverine (●), IBMX (♦) or db-cAMP (■) on the loss of chemotaxis in pregametes of RNAi20 that were shifted into white light at time 0. Compounds were added to pregametes 10 min prior to start of illumination. The concentrations used were 1 μM for chelerythrine, 50 μM for papaverine, 50 μM for IBMX, 20 nM for staurosporine and and 200 μM for SC-9. Non-treated cells (●) were used as control. The fluence rate was 60 µmol m^−2^ s^−1^.

Treatment of pregametes with two phosphodiesterase inhibitors, papaverine and IBMX, assumed to result in elevated levels of cAMP, switched off chemotaxis towards nitrite more rapid than in untreated cultures and similar to those observed for the parental strain (Figure 5B). An increase in cAMP levels as a result of phosphodiesterase inhibition by papaverine and IBMX is known to activate protein kinase A (PKA) [15]. An involvement of cAMP in the control of chemotaxis is supported by the observation that the feeding of db-cAMP caused a faster switching off of chemotaxis than that seen without treatment (Figure 4A and Figure 5B). These results suggest that a PKC and a PKA, targeted by staurosporine and compounds that elevate cAMP levels, respectively, may be components of the same signaling pathway that is involved in the control of the switch-off of chemotaxis to nitrite via phototropin-perceived blue light. The same results were obtained also for effects of these pharmacological compounds on the kinetics of changes in chemotaxis to nitrate (Figure 6).

**Figure 7:**
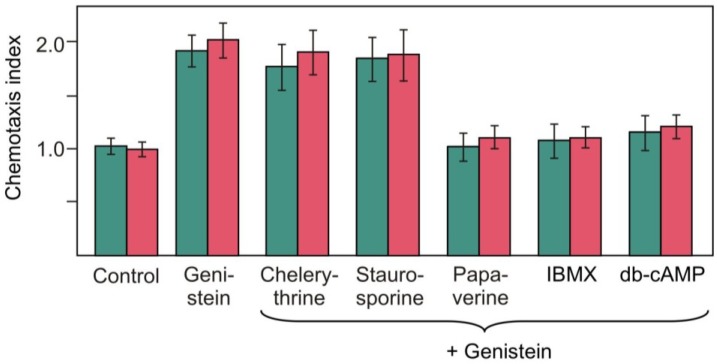
Treatment of pregametes of strain RNAi20 in the light with combinations of pharmacological compounds that either activate or inhibit the loss of chemotaxis in pregametes towards nitrite (■) and nitrate (■). Compounds were added to pregametes 10 min prior to start of illumination at the fluence rates of 60 µmol m^−2^ s^−1^ for 2 h. The genistein concentration was 200 μM. The concentrations used for other compounds were the same as those given in the legend of Figure 5. Non-treated cells (●) were used as control.

We next tested genistein, a protein Tyr kinase inhibitor that negatively regulated the loss of chemotaxis to ammonium in the light [24]. Addition of genistein to pregametes of RNAi20 completely prevented the light-induced switch-off of chemotaxis to nitrite (Figure 7). We assume that phototropin and protein kinases are components of a linear signaling pathway that is involved in the control of the switch-off of chemotaxis.

In order to gain information on the sequence of the protein kinases in this signaling pathway, we employed a combined application of the activators and inhibitors defined above. Since chelerythrine and staurosporine activated loss of chemotaxis in RNAi20, we used these compounds in combination with genistein that prevented loss of chemotaxis to nitrite in the light (Figure 7). Genistein completely blocked the activation seen with PKC inhibitors chelerythrine and staurosporine. This implies that the target of genistein in the signaling pathway is located downstream from the PKC. Genistein, however, did not prevent the switch-off of chemotaxis to nitrite promoted by addition of papaverine, IBMX or db-cAMP in pregametes. This indicates that a cAMP-targeted PKA operates downstream from the PTK inactivated by genistein. The effects were registered only when the assays were performed within 6 h after removal of the nitrogen source from the medium. No effect of the inhibitors on chemotaxis to nitrite in vegetative cells was observed. The effects monitored in pregametes of RNAi20 thus appear not to be caused by interference of these compounds with the signaling mechanism of chemotaxis (data not shown). 

We were also interested in determining whether this signaling pathway is identical to that for the control of blue-light-mediated loss of chemotaxis to nitrate (Figure 7). Taken together these studies suggest that (i) a PKC-like component with a role as negative regulator is involved in the control of chemotaxis to nitrite/nitrate; (ii) a PTK transduces the signal downstream from the PKC-like component; (iii) a PKA transduces the signal downstream from the PTK.

## 3. Discussion

Ammonium is the preferred nitrogen source and chemoattractant for the unicellular green alga *Chlamydomonas reinhardtii*. In the absence of ammonium, vegetative cells can utilize nitrate and nitrite. In *C. reinhardtii* nitrate is also a signalling molecule that controls chemotactic behaviour [17]. Here we have shown that vegetative cells of *C. reinhardtii* sense nitrite and are attracted by this anion as well (Figure 1). Potassium did not act as an attractant for motile cells. 

In an effort to identify if components of nitrate transport/assimilation are required for chemotaxis to nitrate/nitrite, the strain CC-124 with defects in nitrate reductase and NIT2 protein was tested. It was found to exhibit normal chemotaxis to nitrite and nitrate (Figure 1). In *Chlamydomonas*, the NIT2 transcription factor, which contains GAF and Leu zipper domains, is required for nitrate regulation of gene expression [25]. The data suggest that chemotaxis events appear to be specific and independent of those involved in nitrate assimilation. This conclusion is supported by the facts, that (1) nitrate competitively inhibits the nitrite chemotactic activity and vice versa (Figure 2) and (2) pregametes, cells generated in the dark, also demonstrated normal chemotactic activity (Figure 3). More recently, in *Arabidopsis*, nitrate transporter NRT1.1 (CHL1) was identified as a nitrate sensor at high nitrate concentrations [26]. It was demonstrated that NO_3_^−^ transport activity is not required for the sensing function of CHL1. There exists a single copy of NRT1 in the *C. reinhardtii* genome [27]; whether or not this NRT1 protein has a transceptor function as proposed in plants or any other function remains to be addressed in *C. reinhardtii*. Nitrate transporters NRT2.1 and NRT2.2 were shown to be key elements for sensing at low nitrate concentrations to activate gene expression for nitrate assimilation [28]. These transporters dependent on NIT2 activation are however dispensable for nitrate/nitrite sensing in chemotaxis.

During sexual differentiation, *C. reinhardtii* changes its chemotactic behavior not only to ammonium and nitrate but also to nitrite (Figure 3). Unlike vegetative cells and noncompetent pregametes, mature gametes did not show chemotaxis to nitrite. This change in chemotactic behavior requires the sequential action of two environmental cues: nitrogen deprivation and light. Thus, cells starved for nitrogen in the dark (pregametes) still exhibit chemotaxis (Figure 3). Irradiation of pregametes causes both a loss of chemotaxis to nitrite and a gain of mating competence. We conclude that chemotaxis of *Chlamydomonas* towards inorganic nitrogen sources is a property of vegetative cells and pre-gametes but not of gametic cells.

We show here that a reduction in phototropin levels led to a delay in the light-induced switch-off of chemotaxis not only to ammonium [18] and nitrate [17] but also to nitrite (Figure 4). By using a strain strongly reduced in phototropin (RNAi20), we discovered a compensation of this defect upon application of compounds that appeared to activate signal flux: the PKC inhibitors (staurosporine or chelerythrine) or the PKA activators (phosphodiesterase inhibitors or db-cAMP) (Figure 5 and Figure 6). These protein kinases are thus proposed to be downstream components of the phototropin-activated signaling pathway that controls the switch-off of chemotaxis to nitrite and to nitrate. In this pathway, a PKC-like component is proposed to act as negative regulator that attenuates transduction of the light signal. Since genistein inhibited the switch-off of chemotaxis to nitrite/nitrate induced by staurosporine or chelerythrine, a PTK appears to be positioned downstream from the PKC-like activity (Figure 7). A PKA may be positioned downstream from this PTK since conditions that resulted in a rise of intracellular cAMP levels, and thus in an activation of PKA, could be shown to promote the switch-off of chemotaxis even when PTK was inhibited by genistein (Figure 7). Thus, this signaling pathway is identical to that proposed for the control of blue-light-mediated loss of chemotaxis to ammonium [24]. 

**Figure 8:**
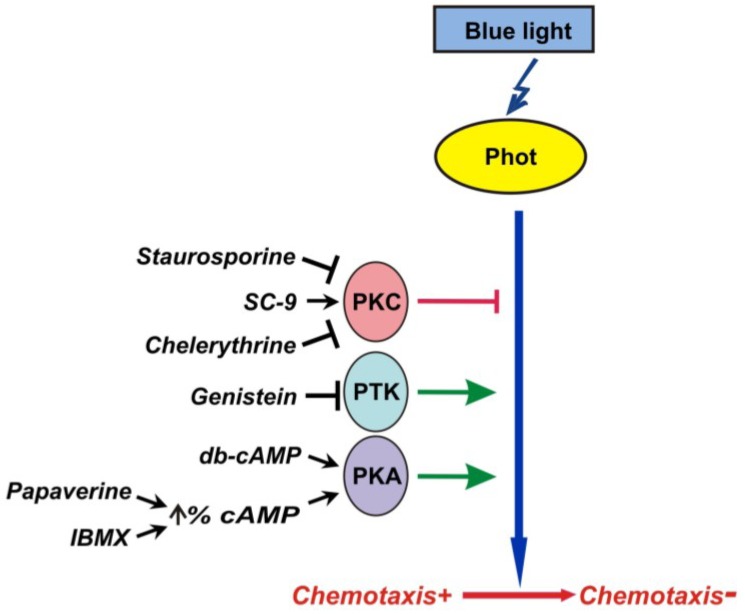
A model for the signaling pathway by which blue light controls chemotaxis towards nitrogen sources in *C. reinhardtii* during pregamete-to-gamete conversion. Black arrows indicate a stimulating effect of pharmacological compounds, T-lines indicate an inhibiting effect. Green arrows indicate stimulation of switch-off of chemotaxis, red T-line indicates exhibiting of switch-off of chemotaxis.

The combined data have been summarized in a model (Figure 8). We assume that at least three protein kinases are components of a linear phototropin-dependent signalling chain controlling the switch-off of chemotaxis. In some respects this blue light signaling pathway shares features with the one required for the formation of sexual competence [29,30]. Both processes employ the same photoreceptor and at least two components, PKC and PKT, of the downstream signalling pathway. Why does *Chlamydomonas* exhibit this intriguing coupling of phototropin-dependent light control for disappearance of chemotaxis towards the nitrogen sources and for gamete formation? It was shown that ammonium and nitrate/nitrite interfered with gametic differentiation [2,10]. Therefore, in gametes, loss of chemotaxis towards nitrogen sources, which are known to play a key role in the repression of the gametic differentiation, may be viewed as one of the cellular adaptations to changing environmental conditions. Seen from this point of view, the coordinated but opposite regulation of chemotaxis towards ammonium, nitrate and nitrite and gamete formation may be of advantage for the alga. 

## 4. Experimental

### 4.1. Strains and Culture Conditions

*Chlamydomonas reinhardtii* strain CC-124 (*nia1, nit2*, *mt^−^*) have been described elsewhere [31]. Another wild-type strain tested was CC-620 *(mt^+^),* obtained from Dr. S. Purton, University College London, GB. Strains RNAi20 and RNAi30 with reduced levels of phototropin [30] were obtained from Dr. C. Beck. Cells were grown at 22 °C under a 12-h light/12-h dark regime in Tris-acetate-phosphate (TAP) medium [32] or in acetate-free TAP (TMP) medium. 

### 4.2. Reagents

All pharmacological compounds were prepared as stocks and stored at −70 °C in the dark. Papaverine was dissolved in water. Other compounds were dissolved in DMSO when an organic solvent was required, or in TAP-N. Papaverine, 3-isobutyl-1-methylxanthine, chelerythrine, genistein, staurosporine, SC-9, and db-cAMP were purchased from Sigma. The components were tested at the concentrations that previously have been shown to be effective in mammalian or plant cell systems (Table 1) and affected pregamete-to-gamete conversion in *C. reinhardtii* [15]. All reagents used did not influence the cellular swimming speed (data not shown).

**Table 1:** Pharmacological compounds that affect light control of chemotaxis.

Compound	Mode of action	Effective concentration	Reference
Papaverine	Phosphodiesterase inhibitor	50 µM	[24,33]
3-isobutyl-1-methylxanthine	Phosphodiesterase inhibitor	50 µM	[15,24]
db-cAMP	PKA activator	15 mM	[34]
Genistein	PTK inhibitor	200 µM	[15,24,35]
Staurosporine	PKA inhibitor	20 nM	[15,36,37]
Chelerythrine	PKC inhibitor	1 µM	[24,38]
SC-9	PKC activator	200 µM	[15,39]

### 4.3. Generation of Pregametes and Gametes

For the generation of pregametes, liquid cultures of synchronously growing cells at the beginning of the light period were washed twice with nitrogen-free medium (TAP-N or TMP-N), resuspended in the same medium at a density of 0.1–1.0 × 10^6^ cells/mL and incubated in the dark for 24 h. Gametes were obtained from pregametes by exposing them to light (fluence rate 60 µmol m^−2^ s^−1^) for 2 h. The light source used was a 500 W lamp (LOMO, Saint-Petersburg, Russia) mounted in a slide projector. 

### 4.4. Determination of Mating Competence

The percentage of gametes was assayed by mixing the cells to be tested with a threefold excess of gametes of opposite mating type. Mating was allowed to proceed in the dark for one hour and stopped by adding glutaraldehyde (final concentration 0.5%). The number of biflagellate cells and quadriflagellate cells in the mating mixture was recorded microscopically. The percentage of mating competent gametes was calculated as described [11]. 

### 4.5. Chemotaxis Assay

Chemotactic responses were tested by counting the number of cells that in darkness swam into rectangular capillaries (260 µm × 450 µm) filled with 3 µL of medium containing either KNO_2_ or the tested source. This number was compared to the number of cells entering capillaries filled with nitrogen-free medium [40]. Capillaries were closed at one end with Parafilm. The other end was submerged in the cell suspension for 10 min at 22 °C. In the competition tests KNO_2_ or KNO_3_ were added to the cell suspension so that the gradient of nitrate or nitrite was generated in the presence of second attractant.

The chemotaxis index (CI) was calculated using the following ratio Equation (1):

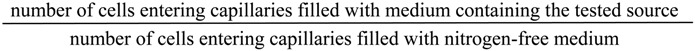
(1)


Switch-off of chemotaxis was recorded as 1.0. Data are means ± SD of triplicate determinations from a representative experiment from at least three different ones.

### 4.6. Protein Isolation, SDS-PAGE and Immunoblot Analysis

*Chlamydomonas* cells (2–4 × 10^6^ cells mL^–1^ in 100 mL) were collected by centrifugation (3000 g, 5 min) and resuspended in 0.1 M DTT, 0.1 M Na_2_CO_3_. Then, 0.66 vol of 5% SDS, 30% sucrose were added. Homogenization of the suspensions was achieved by rapid shaking at room temperature for 20 min. The protein concentration was determined by staining with amido black, using BSA as a standard [41]. After separation of the proteins by SDS-PAGE on a 12% polyacrylamide gel [42], they were transferred to nitrocellulose membranes (Carl Roth, Karlsruhe, Germany) by a semidry blotting (Trans-blot SD BioRad). Blots were blocked in 5% milk in Tris-buffered saline solution with 0.1% Tween 20 prior to 1 h of incubation in the presence of primary antibodies. For this assay, an antibody directed against Chlamydomonas phototropin [6] was used. For a loading control, an antibody that reacts with the co-chaperone CGE1 [43] was employed. The dilutions of the primary antibodies used were as follows: 1:5,000 anti-Phot and 1:6,000 anti-α-CGE1. A 1:10,000 dilution of horseradish peroxidase-conjugated anti-rabbit serum (Sigma) was used as a secondary antibody. The peroxidase activity was detected by an enhanced chemiluminescence assay (Roche). 

## 5. Conclusions

We have shown that vegetative cells of *Chlamydomonas reinhardtii* are attracted by nitrite. The data suggest that chemotaxis events appear to be specific and independent of those involved in nitrate/nitrite assimilation. Chemotaxis towards inorganic nitrogen sources is a property of vegetative cells and pregametes but not of gametic cells. Absorption of blue light by phototropin results in the activation of a signaling pathway that controls the switch-off of chemotaxis. These studies also revealed individual elements of the signal transduction pathway involved in the blue-light-controlled change in the chemotaxis mode of *C. reinhardtii* during gamete formation: three protein kinases, one operating against signal flux and two that promote signal transduction.
